# A Design of Experiments Approach to the Radical-Induced Oxidation of Dimeric C4-C8 Linked B-Type Procyanidins

**DOI:** 10.3390/molecules30010111

**Published:** 2024-12-30

**Authors:** Annik Fischer, Recep Gök, Tuba Esatbeyoglu

**Affiliations:** 1 Department of Molecular Food Chemistry and Development, Institute of Food and One Health, Leibniz University Hannover, 30167 Hannover, Germany; fischer@foh.uni-hannover.de; 2 Institute of Food Chemistry, Technische Universität Braunschweig, 38106 Braunschweig, Germany; r.goek@tu-braunschweig.de

**Keywords:** proanthocyanidin, polyphenol, A-type dimer, B-type dimer, DPPH (2,2-diphenyl-1-picrylhydrazyl) radical, quinone methide, oxidation, synthesis

## Abstract

This study systematically investigated the DPPH (2,2-diphenyl-1-picrylhydrazyl) radical induced oxidation of all dimeric C4-C8 linked B-type procyanidins (PCs) B1–B4 to maximise the formation of the oxidation products using a Design of Experiments (DoE) approach. The C4*β*-C8 linked B1 and B2 formed the A1 (**1**) and A2 (**2**) (*m*/*z* 575 [M-H]^−^) with an ether bridge between C2u-O-C7t as expected. Interestingly, the oxidation of the C4*α*-C8 linked dimers B3 and B4 yielded for each two main oxidation products with *m*/*z* 575 [M-H]^−^. One of them required only a short reaction time (10.0 min, 25.0 °C for B3 (**3**) and B4 (**5**)), whereas the other was maximally formed at a longer time and higher temperature (314 min and 75.0 °C for B3 (**5**); 360 min, 53.7 °C for B4 (**6**)). The formation rates were optimised to 47.4 ± 1.14% (A1; **1**), 27.5 ± 0.76% (A2; **2**), 48.6 ± 4.01% (**3**), 32.0 ± 1.14% (**4**), 45.0 ± 5.14% (**5**) and 60.2 ± 3.68% (**6**).

## 1. Introduction

**Figure 1:**
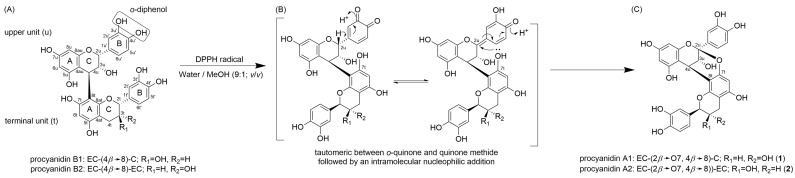
Chemical structures of the C4*β* → C8 linked dimeric B-types procyanidin B1 and B2 (**A**), the formation mechanism according to Kondo et al. [15] (**B**) and the structure of the formed A-type procyanidins A1 (**1**) and A2 (**2**) (main oxidation products, (**C**)).

Procyanidins (PCs) are flavonoids and belong to the group of proanthocyanidins, which are found in a variety of plant-based foods, mainly in fruits (apple, grape), beans (black/red bean), nuts (hazelnut), beverages (milk chocolate, tea, red wine) and spices (cinnamon) [1]. PCs are oligomers and polymers of the flavan-3-ol units (+)-catechin (C) and (–)-epicatechin (EC). In the case of dimeric B-type PCs, the flavan-3-ol units are linked through an interflavan bond, either between the C4 → C8 (B1 to B4; Figure 1A) or between C4 → C6 (B5 to B8). Moreover, the stereochemistry of the interflavan bond varies among the dimers. In particular, the dimers B1, B2, B5 and B7 exhibit a *β*-orientation (4R-configuration) of the C4, whereas the C4 of dimers B3, B4, B6 and B8 has an *α*-orientation (4S-configuration) [2,3]. B-type PCs occur in various fruits such as chokeberries, grapes, and apples [1,4,5]. In addition to the B-type PCs, the A-type PCs contain an ether bridge between C2 of the upper unit (u) and the oxygen of C7 or C5 of the terminal unit (t) (Figure 1C) [3]. Several studies have shown the health benefits of A-type PCs. They are particularly known for their antioxidant activities [6,7], anti-adherence properties against pathogenic bacteria [8], hypoglycaemic activities [9] as well as anti-inflammatory and anti-proliferative activities [10]. A-type dimers can be obtained naturally, for example, from *Litchi chinensis* pericarp and *Arachis hypogaea* (peanut) shells, but in small amounts [4,7,11].

The low occurrence of A-types in nature requires innovative strategies for their large-scale production. Until now, only a few approaches are known. Effective methods include synthesis with benzopyrylium salts and (+)-catechin, with yields between 70–80% [12]. The oxidation of B-type PCs to the A-type is another primary conversion method. Several variations have been described, like basic catalysed reactions with oxygen or hydrogen peroxide [13,14]. Under neutral conditions, Kondo et al. [15] reported the conversion of B1 and B2 into A1 and A2 PCs by DPPH radicals. Furthermore, Osman and Wong [16] observed the A-type conversion by enzymatic oxidation with laccase. Chen et al. [17] used the trimeric B-type PC C1 to convert these into the corresponding A-type at different temperatures, pH values, and catalysts. The catalyst variation was carried out with DPPH radical and superoxide anion radical (radical catalysts). Furthermore, polyphenol oxidase and xanthine oxidase were used as enzymatic catalysts. The results showed that the reaction proceeds chiefly under neutral conditions and that the temperature has a significant effect on the formation rate. The catalyst variation showed no significant difference after a reaction time of 180 min. An average conversion rate of approximately 34% was achieved [17]. Additionally, Jing et al. [18] conducted the DPPH radical-induced conversion of trimeric and tetrameric B-types to obtain the corresponding A-types.

Hayashi et al. [19] described the oxidation of B3 with CuCl_2_ (2.3 mmol/L), resulting in the formation of spirocyclisation products under oxidative conditions. In this study, these products were associated with the colour-deepening phenomenon in aged red rice samples [19]. Moreover, Hibi and Yanase [20] oxidised the trimeric procyanidin C2 and dimer B3 by using CuCl_2_ and detected the generation of various spirocyclisation products. Here it was described that due to the 4S configuration at the C4 atom, no ether bridge C2u-O-C7t (A-type PC) was formed [20]. Kataoka et al. [21] also reported that the *α*-orientation of the interflavan bond has an influence on the oxidation products, and also here spirocyclisation products of B4 were formed by using CuCl_2_ asan oxidant. The oxidation of B2 with CuCl_2_ was conducted by Suwa and Yanase [22]. Here the oxidation of procyanidin B2 to A2 was not observed, which contrasts with the results of the studies by Kondo et al. [15] (DPPH-induced oxidation) and Osman and Wong [16] (laccase-catalysed oxidation). Suwa and Yanase [22] suggest that the acidic conditions present in the CuCl_2_-water solution inhibit the formation of the A-types during oxidation.

Thus, there are only a few studies on the oxidation of B3 and B4 and these have not optimised the reaction conditions of the main oxidation products for their targeted isolation. Optimisations in this area are often carried out using the one-factor-at-a-time (OFAT) method. In this method, the values of the individual factors are varied stepwise, while keeping the levels of the other factors constant. This will only give a good result if the factors do not interact with each other [23]. Therefore, the formation rates in the current study were maximised based on the DoE. This experimental design has the best ratio between the number of experiments and the amount of information that can be obtained [23]. Here, a Box–Behnken design (BBD) was chosen to study the radical-induced oxidation because it is slightly more efficient in terms of the number of experiments and power than a central composite design (CCD) [24].

There are currently no approaches for the systematic investigation of the oxidation of all C4 → C8 linked dimeric PCs, especially the oxidation of the dimers B3 and B4 (C4α → C8 linked PCs) with DPPH radicals, which has not been described in the literature. A number of studies have investigated the health-promoting effects of the oxidation products A1 and A2, which are obtained from B1 and B2 [25,26,27,28]. The oxidation products of B3 and B4 are also assumed to have a health beneficial effects, but this has not yet been investigated. Therefore, it is important to optimise these oxidation products, as well as A1 and A2, in order to isolate them on a larger scale and to determine their structure–activity relationship (SAR).

## 2. Results and Discussion

In this study, the C4*β* → C8 linked dimers (B1, B2), and the C4*α* → C8 linked dimers (B3, B4) were oxidised with DPPH radicals under the same conditions. The reaction temperature, reaction time and the ratio of B-type to DPPH radical (*n*/*n*) were optimised using a BBD (Table 1) in order to obtain maximum yields of the main oxidation products (*m*/*z* 575 [M-H]^−^) from B1–B4. The systematic optimisation of all C4 → C8 linked dimers revealed that the configuration has a significant influence on the formation of the oxidation products.

**Figure 2:**
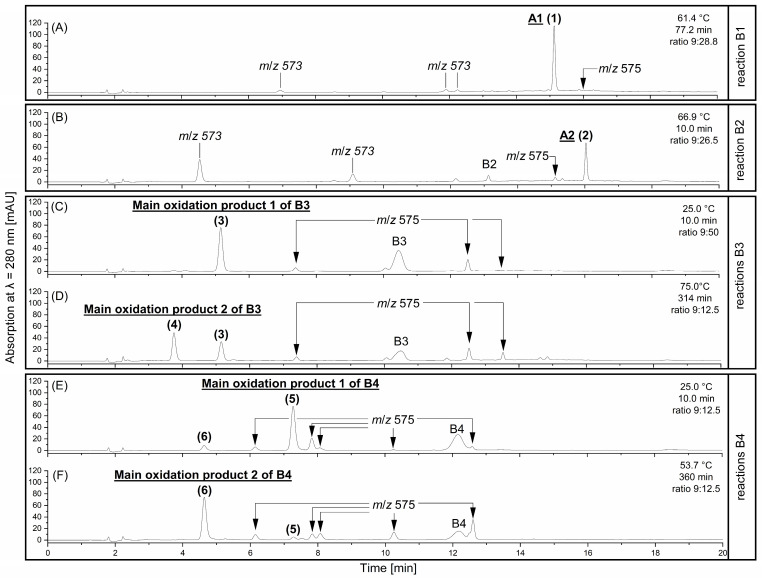
HPLC-PDA chromatograms at λ = 280 nm, obtained under the optimal conditions for the main oxidation products **1**–**6** with *m*/*z* values in negative mode (**A**) Oxidation of B1 was carried out at 61.4 °C for 77.2 min, with a ratio of B-type to DPPH radical of 9:28.8 (*n*/*n*) for compound **1**. (**B**) Oxidation of B2 was performed at 66.9 °C for 10.0 min, with a ratio of B-type to DPPH radical of 9:26.5 (*n*/*n*) for compound **2**. The C4α-C8 linked B3 and B4 showed two peaks with *m*/*z* 575 [M-H]^−^ and a high formation rate about 20%. Therefore, both compounds were optimised (C/D and E/F). (**C**) Oxidation of B3 at 25.0 °C for 10.0 min, with a ratio of B-type to DPPH radical of 9:50 (*n*/*n*) for compound **3**. (**D**) Another oxidation of B3 was performed at 75.0 °C for 314 min, with a ratio of B-type to DPPH radical of 9:12.5 (*n*/*n*) for compound **4**. (**E**) Oxidation of B4 carried out at 25.0 °C for 10.0 min, with a ratio of B-type to DPPH radical of 9:12.5 (*n*/*n*) for compound **5** and (**F**) at 53.7 °C for 360 min, with a ratio of B type to DPPH radical of 9:12.5 (*n*/*n*) for compound **6**.

The oxidised B1 and B2 solution showed one main oxidation product with *m*/*z* 575 [M-H]^−^ expected as A1 (**1**) and as A2 (**2**) according to Kondo et al. [15]. The HPLC-PDA chromatograms after DPPH radical-induced oxidation of B1 (Figure 2A) and B2 (Figure 2B) showed that under the optimised reaction conditions, mainly compounds **1** and **2** were produced.

The HPLC-ESI-MS^n^ analysis of the reactions obtained from the B-types with an C4*β* → C8 interflavan bond confirmed the characteristic fragmentation pattern of the A-type PCs (Figure S1A and Tables S1 and S2, Supplementary Materials). Here, the characteristic heterocyclic ring fission cleavage (HRF; loss of −126 Da) with a product ion of *m*/*z* 449, the retro Diels-Alder cleavage with *m*/*z* 423 (RDA, −152 Da) and the quinone methide (QM) cleavage with *m*/*z* 285 and *m*/*z* 289 were detected (Figure S2A, Supplementary Materials) [29,30]. Furthermore, commercial analytical standards of A1 and A2 as authentic reference samples were used to prove that the compound **1** (retention time R_t_ = 15.2 min; Figure 2A) and the compound **2** (R_t_= 16.1 min; Figure 2B) were the expected A1 and A2. Based on the same UV-spectrum, retention time on HPLC and MS/MS fragmentation, the identification of these compounds as A1 and A2 can be confirmed. The conversion of B1 and B2 can be explained by the 1,6-intermolecular nucleophilic addition mechanism, involving a quinone methide intermediate (Figure 1B), resulting in the formation of A1 and A2 [15,17].

A1 (**1**) and A2 (**2**) elute after the corresponding B-type B1 (R_t_ = 9.8 min) and B2 (R_t_ = 13.1 min). This signifies that these A-type products have a decrease in polarity compared to the B-types. This is expected due to the formation of the ether bridge between the O7 of the terminal unit (t) and the C2 of the upper unit (u) (Figure 1B). Kondo et al. [15] and De Taeye et al. [31] also reported a higher interaction of the B-types B1 and B2 than the corresponding A-type with an RP18 HPLC column, resulting in earlier elution of the B1 and B2 than A1 and A2.

Moreover, the formation of further oxidation products with *m*/*z* 573 [M-H]^−^ was also observed (Figure 2A,B). We assume that these products are structurally analogous to the isolated oxidation products with *m*/*z* 573 [M-H]^−^ of Suwa and Yanase [22] formed in the oxidation of B2 with CuCl_2_.

**Figure 3:**
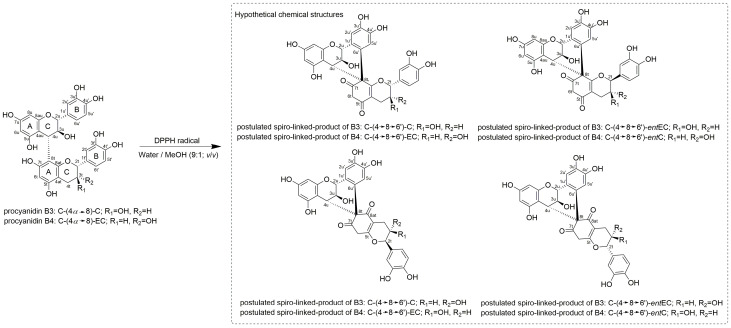
Chemical structures of the C4*α* → C8 linked B-types B3 and B4 and the hypothetical chemical structures of the oxidation products from B3 and B4 after the reaction with DPPH radicals based on the isolated compounds of Hayashi et al. [19] and Kataoka et al. [21].

In the DPPH radical-induced oxidation of B3 and B4, a large number of oxidation products with *m*/*z* 575 [M-H]^−^ were observed (Figure 2C–F). Two oxidation products with *m*/*z* 575 [M-H]^−^ exhibited significantly higher formation rates than the others and represent the main oxidation products. These products are referred to as oxidation products 1 (**3**, **5**) and 2 (**4**, **6**) of B3 and B4 (Figure 2C–F). The HPLC-ESI-MS^n^ analysis of these products showed the same HRF- (*m*/*z* 449 [M-H-126 Da]^−^) and RDA-cleavage (*m*/*z* 423 [M-H-152 Da]^−^) as A1 (**1**) and A2 (**2**) (Figure S1A,B; Tables S3 and S4, Supplementary Materials). However, a specific difference in the fragmentation pattern of the oxidation products **3** and **4**, which is the typical QM fragment *m*/*z* 287, indicates that spirocyclisation products (Figure 3) instead of products with a C2u-O-C7t ether bridge were formed in this work, as observed in the CuCl_2_-induced oxidation of B3 and B4 [19,20,21]. The QM cleavage patterns are demonstrated in the supplementary material in Figure S2A,B. The spirocyclisation products described by Hayashi et al. [19] and Kataoka et al. [21] are characterised by a C4u-C8t-C6′u bond structure. Here, the formation of a new pyran ring can occur with the participation of C5t and further products may be formed (Figure 3) [19,20,21]. The spiro-linked-compounds **3**–**6** were probably formed by a 1,4-intramolecular nucleophilic addition of a terminal unit of the A-ring to the *o*-quinone form of the B-ring (upper unit) based on Hayashi et al. [19] and Kataoka et al. [21]. The detailed NMR characterisation of the latter main oxidation products of the DPPH radical oxidation and the determination of the SAR are the subject of our ongoing research.

### 2.1. Investigation of the Formation of the A-Type PCs from C4β → C8 Linked B1 and B2

Dimers B1 (EC-(4*β* → 8)-C) and B2 (EC-(4*β* → 8)-EC) showed similar behaviour in the formation of the ether bridge by 1,6-intramolecular nucleophilic addition caused by DPPH radicals in terms of reaction products and oxidation parameters (temperature, time, ratio B-type to DPPH). The empirical models for the percentage formation rate of the A-type products obtained from the experimental data set according to the BBD are shown in Table 2.

**Figure 4:**
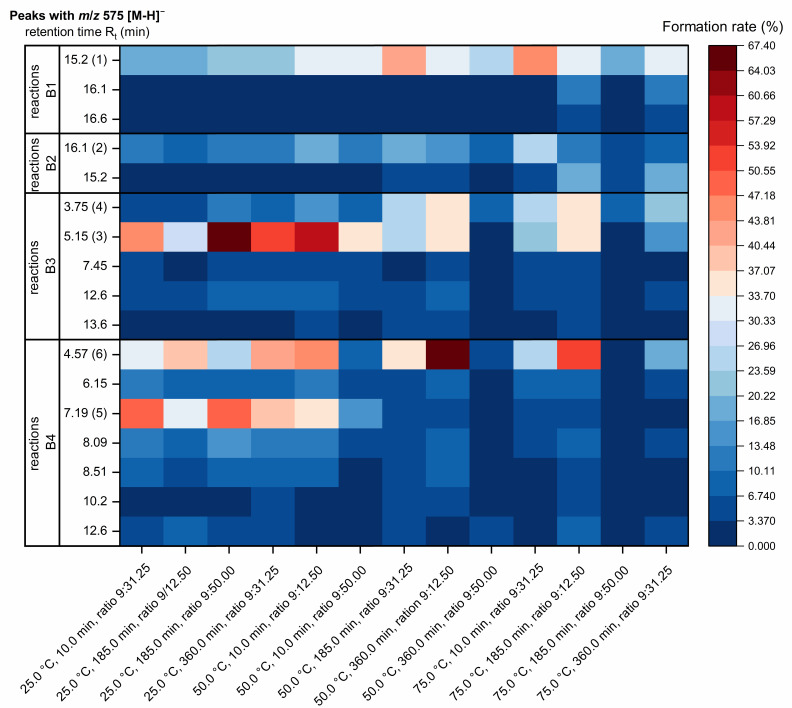
Heatmap of the formation rate [%] of *m*/*z* 575 [M-H]^−^ products from B1 to B4 using the reaction parameters applied for the Box-Behnken design determined by HPLC-PDA at λ = 280 nm.

Under two specific BBD conditions, the reactions of B1 exhibit the formation of two additional products (R_t_ = 16.1 min and 16.6 min) with *m*/*z* 575 [M-H]^−^ in amounts of >3.4% (Figure 4). Dimer B1 forms one main product (*m*/*z* 575 [M-H]^−^) with a retention time of 15.2 min (**1**) and a formation rate between 15 and 45%. For B2, an additional oxidation product (R_t_ = 15.2 min) was formed over an amount of >3.4% by half of the BBD conditions (Figure 4). The main oxidation product (**2**) (*m*/*z* 575 [M-H]^−^) was obtained at a retention time of 16.1 min from B2 with a formation rate ranging from 5 to 32%. For both B1 and B2, the formation of other *m*/*z* 575 [M-H]^−^ products can only be observed by high reaction temperatures (75.0 °C) and extended reaction times (185.0 min and 360.0 min) with small formation rates (Figure 4). Under optimum formation conditions of 61.4 °C, 77.2 min, ratio B-type to DPPH radical of 9:28.8 (*n*/*n*) for B1 and 66.9 °C, 10 min, ratio B-type to DPPH radical of 9:26.5 (*n*/*n*) for B2, only minimal amounts of less than 2.5% of other oxidation products with *m*/*z* 575 [M-H]^−^ were formed (Figure 2A,B).

The formation of isomeric A-type products with *m*/*z* 575 [M-H]^−^ was expected, since it is also known that epimerisation of the PC monomers (flavan-3-ols) takes place at 80 °C [32]. Therefore, it is conceivable that the epimerisation could also occur at higher temperatures on the C-ring of the terminal unit. Poupard et al. [30] described during the oxidation of B2 in addition to A2 a further isomer with *m*/*z* 575 [M-H]^−^. Here it was assumed that a product with an ether bridge between C6′t-*O*-C5u or C2′t-*O*-C5u was formed [30].

**Figure 5:**
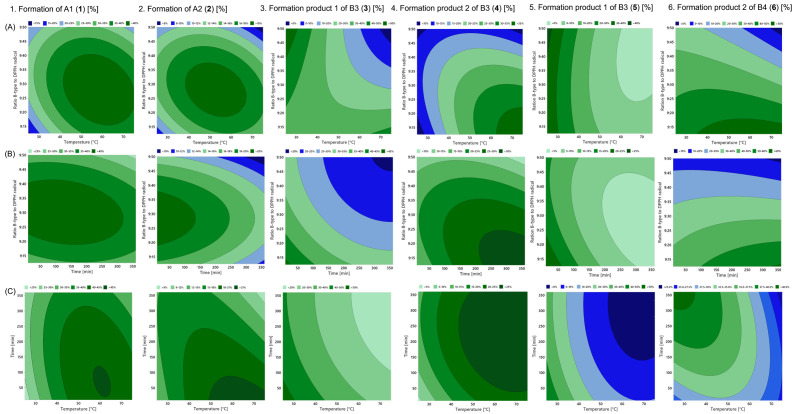
Contour plots of the main oxidation products **1**–**6** from B1 (**1**), B2 (**2**), B3 (**3**, **4**) and B4 (**5**, **6**). (**A**) Contour plot of the interaction between the ratio of dimeric B-type procyanidins to DPPH radicals and the reaction temperature. (**B**) Contour plot of the ratio of B-type PC to DPPH radicals and the reaction time. (**C**) Contour plot of reaction time and reaction temperature.

The empirical models generally show a good fit to the experimental data. The R^2^ for the formation of A1 (**1**) is 91.0% and for A2 (**2**) is 85.5% (Table 2). The square of the ratio of B-type to DPPH radical (x_3_^2^), as well as the square of the reaction temperature (x_1_^2^) for B1, and the time (x_2_) for B2 have the most significant effect on the formation of the PCs A1 (**1**) and A2 (**2**) (Figure S3 Supplementary Materials). The contour plots in Figure 5A1–C1,A2–C2 expose the interaction between the ratio of B-type to DPPH radical and the reaction temperature as well as the reaction time and the interaction between temperature and time for the formation rate of the A-type products. It is evident that the interactions between each factor (x_1_, x_2_, x_3_) are quite equal. The maximum formation is for both in the centre of the reaction space (Figure 5A1,A2). The optimal reaction conditions with the maximal percentage of the formation rate are discussed in Section 2.3. The highest temperature of 75.0 °C in the reaction space was chosen so that the formation of A1 and A2 is in the middle of the plot, but not much higher, so that the formation of isomers is not too high.

### 2.2. Investigation of the Formation of the Oxidation Products from C4α → C8 Linked B3 and B4

The results of the DoE study demonstrates clearly that C4*α* → C8 linked dimeric PCs B3 and B4 do not react optimally under the same conditions to the main oxidation products as the C4*β* → C8 linked dimeric PCs B1 and B2 (Figure 5). Moreover, they generate a greater variety of isomeric products with *m*/*z* 575 [M-H]^−^ at low concentrations than B1 and B2 (Figure 4). The oxidation product 1 (**3**, **5**) and 2 (**4**, **6**) of B3 and B4 demonstrated significantly higher formation rates compared to the others, with a strong dependence on the reaction conditions and were therefore optimised (Figure 4, Table 3).

The oxidation product 1 of B3 (**3**; R_t_ = 5.15 min), was formed optimally at 25.0 °C, at a short reaction time of 10 min and a B-type to DPPH radical ratio of 9:50.0 (*n*/*n*). The reaction requires only a minimal amount of external energy and a short reaction time. On the other hand, the maximum formation of the oxidation product 2 of B3 (**4**; R_t_ = 3.75 min) occurred at 75.0 °C, with a longer reaction time of 314 min and a ratio of B-type to DPPH radical of 9:12.5 (*n*/*n*) (Table 3). Here, a higher energy input is necessary for the formation of these products. We assume that product 2 of B3 (**4**) and B4 (**6**), which are formed under higher energy input, are spirocyclisation products of the corresponding B-type, either with different stereochemistry or rearrangement products in which a new pyran ring is formed with the participation of C5t (Figure 3). Hayashi et al. [19] and Kataoka et al. [21] described that further recycling of a quinone methide at the B-ring of the terminal unit results in the loss of the stereochemistry at C2t and the formation of the other enantiomer.

The regression of the empirical model for the oxidation product 1 of B3 (**3**) has a low correlation with the experimental data set, with 78.1% (despite n = 4; Table 2). The model for the oxidation product 2 of B3 (**4**) with the same experimental data set shows an R^2^ of 93.5%. We suggest that the oxidation product 1 of B3 might react further, so the individual biological replicates cause a high scatter, which can explain the R^2^ of 78.1%.

The oxidation product 1 of B4 (**5**; R_t_ = 7.19 min; Figure 2E) was formed maximally at 25.0 °C, at a reaction time of 10.0 min and a B-type to DPPH radical ratio of 9:12.5 (*n*/*n*) (Table 3). On the other hand, the oxidation product 2 of B4 (**6**; R_t_ = 4.57 min), similar to the product 2 of B3 (**4**), was maximally formed at high temperatures (53.7 °C), long reaction times (360.0 min) and with a ratio of B-type to DPPH radical of 9:12.5 (*n*/*n*) (Table 3).

A comparison of the contour plots of the formation of the oxidation products 1 and 2 suggests that their reaction behaviour is not very similar compared to that of A1 (**1**) and A2 (**2**) (Figure 5). The interaction between the ratio of B-type to DPPH radicals and the reaction temperature for the formation of the oxidation product 1 (**3**, **5**) shows that low temperatures are the significant factor for the formation of these products (Figure 5A3,A5; Figure S3, Supplementary Materials). In contrast, high temperatures and long reaction times promote the formation of the products 2 (**4**, **6**) with a lower amount of DPPH radicals (Figure 5A4,A6,B4,B6).

The retention times on an RP18 phase indicate that the oxidation products 1 (**3**, **5**) have a stronger interaction with the stationary phase compared to the oxidation products 2 (**4**, **6**) (Figure 2C–F). Furthermore, the oxidation products (**3**–**6**) interact less with the stationary phase than B3 and B4, suggesting an increase in polarity of the oxidised products, but the formation of the spiro-linked-products as well as oxidation decrease the polarity, so we assume that the differences in retention time are due to the change in three-dimensional structure. Further isolation and structural elucidation are required to provide more specific information.

### 2.3. Optimisation of the Percentage Formation Rate of the Oxidation Products

**Figure 6:**
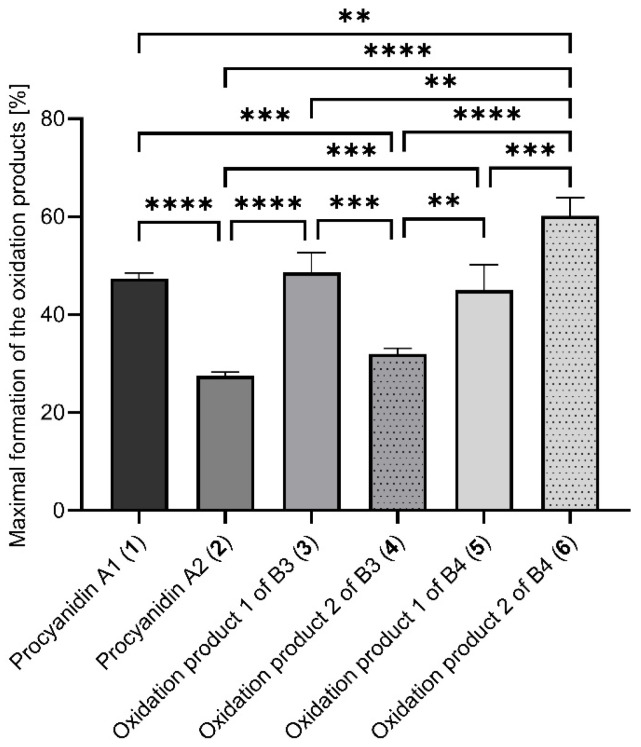
Formation rate of the main oxidation products **1**–**6** [%] under the optimised reaction conditions of B1 to B4. The formation rate values are expressed as mean ± standard deviation from three biological replicates. Statistical differences between the different formation rates of the oxidation products were determined by one-way ANOVA followed by Tukey’s multiple comparison test, ** *p* < 0.01; *** *p* < 0.001; **** *p* < 0.0001. Non-significant associations are not shown.

To determine the optimum reaction parameters for the maximal formation of the oxidation products, a target size optimisation was performed using the empirical models of the different compounds **1**–**6** (Table 2). For this purpose, a maximisation of the regression equation was carried out to determine the levels of the factors x_1_, x_2_, and x_3_ for the maximum formation rate for each compound (**1**–**6**). The reaction conditions for the maximal formation of the different products are given in Table 3. The formation rates of the products **1**–**6**, predicted by the model and determined experimentally, indicate significant differences between the various oxidation products, which can be attributed to structural differences. The radical-induced oxidation of the C4*β* → C8 linked B1 shows a rate of formation of 47.4 ± 1.14%. This means almost 50% of B1 is oxidised to A1 (**1**). This shows that B1 has a higher A-type formation rate (*p* < 0.0001) than the other C4*β* → C8 linked dimer B2 (Figure 6). B2 achieved a formation rate of 27.5 ± 0.76% for A2 (**2**) (Table 3). We assumed that the higher formation rate of A1 (**1**) than A2 (**2**) is due to the 2,3 *cis*-configuration of the terminal unit of B2, which enhances the formation of compounds with *m*/*z* 573 [M-H]^−^ (Figure 2A,B), as observed in Suwa and Yanase [22] during the oxidation of B2 with CuCl_2_.

Interestingly, for B3 (C-(4*α* → 8)-C) and B4 (C-(4*α* → 8)-EC), no significant amounts of compounds with *m*/*z* 573 [M-H]^−^ were detected (Figure 2C–F). This suggests that these products may be less susceptible to other intramolecular nucleophilic addition.

In addition, it was observed that the formation rate of the oxidation product 2 (**4**) was notably lower (*p* < 0.001) than the formation of product 1 (**3**) obtained from B3, while the oxidation product 1 of B4 (**5**) was significantly lower (*p* < 0.001) than the product 2 of B4 (**6**) (Figure 6). The low experimental formation rate of the product 2 of B3 (**4**) with 32.0 ± 1.14% compared to the other oxidation products of B3 and B4 (**3**, **5**, **6**; Figure 6) can be attributed to the formation of the product 1 (**3**) under the optimised reaction conditions of the product 2 of B3 (**4**) (Figure 2D).

Overall, the oxidation product 2 of B4 (**6**) showed the best formation rate with 60.2 ± 3.68% of all C4 → C8 linked B-types (*p* < 0.01) followed by the A1 (**1**) (47.4 ± 1.14%) and the A-type product 1 of B3 (**3**) with 48.6 ± 4.01% (Figure 6). Altogether, the stereochemistry of the C4 → C8 linkage does not substantially influence the maximum rate of the oxidation product formation (Figure 6, Table 3).

## 3. Materials and Methods

### 3.1. Chemicals

Ultrapure water was prepared with the Purelab^®^ flex 3 from Elga Veolia (Celle, Germany). Acetonitrile UHPLC supergradient (>99.9%) was acquired from PanReac AppliChem ITW Reagents (Darmstadt, Germany). Acetonitrile LC-MS grade (>99.9%) was obtained from Honeywell Riedel-de Haën^TM^ (Seelze, Germany). Acetic acid, LC-MS grade, was purchased from Honeywell Fulka^TM^ (Seelze, Germany). Acetic acid (Rotipuran^®^, 100%) and methanol (Rotisolv^®^, HPLC >99.9%) were acquired from Carl Roth (Karlsruhe, Germany). (+)-Catechin hydrate (≥98%, HPLC) and (–)-epicatechin (≥90%, HPLC) were purchased from Sigma–Aldrich (Steinheim, Germany). 2,2-Diphenyl-1-picrylhydrazyl (DPPH, free radical with 95%) was obtained from abcr (Karlsruhe, Germany). Procyanidine A1 and A2 were purchased from Extrasynthese (Lyon, France).

### 3.2. HPLC-PDA Analysis

The analysis of the different reactions was performed on an Agilent HPLC 1290 series (Waldbronn, Germany) equipped with a G7120A high-speed pump, a G7167B multisampler and a G7117B PDA detector. The stationary phase was an Aqua C18, 3 µm, 125 Å, 150 × 2 mm column with a guard column (Phenomenex, Aschaffenburg, Germany). Two percent aqueous acetic acid (*v*/*v*) and acetonitrile (HPLC grade) were used as solvents A and B, respectively. The elution was carried out on gradient mode at 25 °C with a flow rate of 0.25 mL/min: 3–10% B (0–9 min), 10–35% B (9–16 min), 35–75% B (16–19 min), 75% B (19–20 min), 75–100% B (20–21 min), and 100% B (21–26 min). Afterwards, a re-equilibration was conducted at 3% B for a period of 5 min. Detection took place at λ = 280 nm. The multisampler temperature was adjusted at 10 °C. PCs were quantified with an external calibration as dimer B1 equivalent (R^2^ = 1; limit of detection = 5.79 ng/mL (LOD), limit of quantification = 1.57 ng/mL (LOQ)).

The percentage formation rate of the oxidation products Equation (1) was determined by the ratio of the concentration of the oxidation product (c_product_) to the applied concentration of B-type (c_B-type_):

(1) $$Formation\;rate\;[\%]=\frac{c_{product}}{c_{B-type}}\times 100$$

### 3.3. HPLC-ESI-MS/MS Analysis

The HPLC analysis was performed on an 1100 series HPLC from Agilent equipped with a G1312A binary pump, a G1329B autosampler and a G1315B PDA detector. The separation was performed with an Aqua C18, 3 µm, 125 Å, 150 × 2 mm column equipped with a guard column (Phenomenex) at 25 °C and a flow rate of 0.25 mL/min. One percent aqueous acetic acid (*v*/*v*; A) and acetonitrile (B) were used as mobile phases. The gradient elution was performed analogous to the HPLC-PDA analysis: 3–10% B (0–9 min), 10–35% B (9–16 min), 35–75% B (16–19 min), 75% B (19–20 min), and 100% B (21–26 min). The re-equilibration to 3% B was extended to 8 min.

The HPLC system was connected to a HCT ultra PTM Discovery system (ESI-Ion Trap MS/MS) from Bruker Daltonics (Bremen, Germany). MS analysis was recorded under the following conditions: negative ionisation mode, capillary +3000 V, capillary exit −500.0 V; skimmer −40.0 V; cap exit −121.0 V; dry gas N_2_ 10.0 mL/min; dry temperature 325 °C; nebulizer 60 psi; scan range *m*/*z* 100–1200.

### 3.4. Preparation of Dimeric B-Type Procyanidins

The preparation and isolation of dimeric PCs were carried out according to Esatbeyoglu et al. [5] and Esatbeyoglu and Winterhalter [33]. *Aronia melanocarpa* pomace and seeds were used to synthesise the C4*β* → C8 linked dimers B1 and B2 with (–)-epicatechin as the upper unit [33]. The C4*α* → C8 linked dimers B3 and B4, with (+)-catechin as the upper unit, were synthesised using *Salix alba* bark [5]. Authentic reference material from Esatbeyoglu et al. [5] and Esatbeyoglu and Winterhalter [33], identified by nuclear magnetic resonance (NMR) spectroscopy and phloroglucinolysis, were used for identification of the different dimers by HPLC-PDA and LC-ESI-MS/MS. Preparative HPLC (Section 3.5) was used after CCC separations to achieve a purity higher than 95% (λ = 280 nm).

### 3.5. Preparative HPLC

Purification by preparative HPLC was carried out on an ECOM HPLC system (Chrášťany u Prahy, Czech Republic) with a preparative pump ECP2050, a four-way gradient valve ECB2007, a UV-detector for four different wavelengths TOY20DAD 800 H, and a 2-channel online degasser A5328 from Knauer (Berlin, Germany). As the stationary phase, a Luna C18, 5 μm, 100 Å, 250 × 21.2 mm column with a guard column (Phenomenex) and as the mobile phase ultrapure water (A) and acetonitrile (B) were used. The separation was performed at 25 °C with a flow rate of 15 mL/min using the following gradient: 3–10% B (0–28 min), 10–25% B (28–50 min), 25–75% B (50–60 min) and 75–100% B (60–70 min). The eluate was fractionated manually according to the UV-chromatogram at λ = 280 nm.

### 3.6. DPPH Radical-Induced Oxidation of B-Type Procyanidins

The radical-induced oxidation was modified according to Chen et al. [17] and Kondo et al. [15]. The conversion was carried out to the BBD plan (Table 1, further information in Section 3.7) with the different coded levels x_1_ = 25.0–75.0 °C, x_2_ = 10.0 min–60.0 min and x_3_ = 9:12.5–9:50.0 (*n*/*n*). For each reaction, 900 μL of a 0.10 mM aqueous B-type solution was mixed with the corresponding volume of a 5 mM methanolic DPPH radical solution according to the BBD coded levels of x_3_ (9:12.50, 9:31.25, 9:50.00; *n*/*n*) and made up to 1 mL with methanol in a 1.5 mL SafeSeal reaction vessel from Sarstedt (Nümbrecht, Germany). The reaction was carried out in a shaking water bath (model 1070, four temperature water baths from GFL, Burgwedel, Germany), which allowed all three temperature levels to be carried out simultaneously. After the appropriate reaction time, the solution was cooled at −20 °C for 10 min to slow down the reaction. Afterwards, the solutions were filtered with a 0.2 μm syringe filter from Wicom (Darmstadt, Germany), followed by HPLC-PDA and HPLC-ESI-MS/MS analysis.

### 3.7. Optimisation of the Radical-Induced Oxidation of B-Type Procyanidins Based on Design of Experiment

For the optimisation of the DPPH radical-induced oxidation, the reaction temperature, reaction time and mass ratio between the B-type and DPPH radicals were selected as factors. Previous experiments with B1 showed that time, temperature and the ratio of B1 to DPPH radicals (*n*/*n*) have a significant effect on the reduction of B1. The optimisation with the BBD was performed for all four C4 → C8 linked B-types. To compare the DPPH radical-induced oxidation process of each B-type, the same level conditions for temperature (x_1_), time (x_2_), and B-type to DPPH radical ratio (x_3_) were used (Table 1). Each factor has three coded levels: −1, 0, and +1. Therefore, the BBD comprises 15 experiments with three central points by using three continuous factors (x_1_, x_2_, x_3_). The trials were always carried out randomised to record the time-related variation of substances during the HPLC-PDA analysis. The biological replicate was considered as one block for the evaluation. In total, 45 experiments were conducted for B1, B2, and B4 (n = 3). The oxidation of B3 was carried out with n = 4 (60 experiments) due to the variation in the formation of the oxidation product 1 of B3 (no outlier according to Grubbs with α = 0.05).

Minitab^®^ Statistical Software version 21.1.0 (Minitab lnc., State College, USA) was used for the analysis of the model. Based on the BBD, a quadratic model (second-order polynomial model) was used for the regression of the empirical model Equation (2):

(2) $$f\left(x\right)=b_{0}+b_{x_{1}}x_{1}+b_{x_{2}}x_{2}+b_{x_{3}}x_{3}+b_{x_{1}x_{2}}x_{1}x_{2}+b_{x_{1}x_{3}}x_{1}x_{3}+b_{x_{2}x_{3}}x_{2}x_{3}+b_{{x_{1}}^{2}}{x_{1}}^{2}+b_{{x_{2}}^{2}}{x_{2}}^{2}+b_{{x_{3}}^{2}}{x_{2}}^{2}$$

The quadratic model contains a linear-, interaction-, and quadratic part. In Equation (2), x_1_, x_2_ and x_3_ are the factors of the empirical model and $b_{x_{i}}$ is the coefficient value estimated by the regression [23]. Furthermore, an analysis of variance (ANOVA) with degree of freedom (df), corrected sum of square (ss), corrected mean of square (ms), F-value and *p*-value (determine significance *α* = 0.05) was performed. If necessary, predictors without a statistically significant association with the response variable were reduced from the model. Also, the R-squared (R^2^) of the regression was determined to compare the variability of the experimental data to the polynomial model.

### 3.8. Target Size Optimisation

An optimisation of the size of the target variables was performed. Therefore, the quadratic model of the BBD was used. The formation of the main oxidation products (**1**–**6**) was the primary response variable. The optimisation of the response variables was performed using Minitab^®^ Statistical Software. The overall desirability was determined for each response variable individually with a weight of one. The resulting variables (x_1_, x_2_, x_3_) with the maximum formation rate were examined experimentally by performing the reaction analogous to Section 3.6.

### 3.9. Statistical Analysis

For statistical analysis, one-way ANOVA followed by Tukey’s multiple comparison test was used to determine statistically significant differences. This was carried out with GraphPad Prism version 10.2.1 (San Diego, CA, USA).

## 4. Conclusions

The systematic investigation of radical-induced oxidation of the C4 → C8 linked B-types using DoE in combination with liquid chromatography and mass spectrometry revealed that the formation of oxidation products of all four C4 → C8 linked B-types were not analogous under the conditions used in the present study. The results showed that the C4*β* → C8 linked dimers B1 and B2 exhibited similar interactions between the ratio of B-type to DPPH radical and the temperature during radical-induced oxidation, as well as a similar temperature–time relationship for the formation of the A-type products (Figure 5). The C4*α* → C8 linked dimers B3 and B4 exhibited two main oxidation products (**3**–**6**), which, based on QM fragmentation, were identified as spiro-linked-compounds, as previously documented in the literature for the oxidation of B3 and B4 [19,20,21]. The maximum formation of one oxidation product isomer required a minimal input of energy (**3**, **5**), while the others maximally formed at longer reaction times and temperatures (**5**, **6**). The contour plots of the ratio of B-type to DPPH radicals and the reaction temperature and time interactions of the products 1 and 2 of B3 and B4, as well as the temperature–time plot, showed the same formation trend (Figure 5). In general, the results indicate that the orientation of the interflavan bond had no effect on the formation rate of the oxidation products, but it did affect the chemical structure of the oxidation products. Further studies are required to perform the reactions on a preparative scale to isolate the products 1 and 2 of B3 and B4 (**3**–**6**) to determine the chemical structure, including stereochemistry by NMR experiments, which is our ongoing research. These results provide additional information for a better understanding of the oxidation processes of PCs, as they may be induced during food processing, and for the optimised synthesis and isolation of A1, A2 and the main oxidation products of B3 and B4 to obtain useful information about the structure–activity relationship of procyanidins on their antioxidant activity.

## Figures and Tables

**Table 1:** Experimental conditions for the Box–Behnken design for the radical oxidation of B-type PCs.

Factor	Symbol	Coded Levels		
		−1	0	+1
Temperature [°C]	x_1_	25.0	50.0	75.0
Time [min]	x_2_	10.0	185.0	360.0
Ratio B-type/DPPH radical (*n*/*n*)	x_3_	9:12.50	9:31.25	9:50.00

**Table 2:** Equation of the formation rates [%] from the individual optimised products **1**–**6** obtained from B1 to B4.

f(x) = Formation of…	Retention Time (R_t_) [min]	Equation of Regression Analysis	R^2^ [%]
procyanidin A1 (**1**) [%]	15.2	f(x) = −65.0 + 2.35x_1_ + 0.0945x_2_ + 0.242x_3_ − 0.01595x_1_^2^ − 0.00010x_2_^2^ − 0.0289x_3_^2^ − 0.00096x_1_x_2_ − 0.0107x_1_x_3_ − 0.00067x_2_x_3_	91.0
procyanidin A2 (**2**) [%]	16.1	f(x) = −24.4 + 1.00x_1_ + 0.0434x_2_ + 1.09x_3_ − 0.00641x_1_^2^ − 0.000032x_2_^2^ − 0.0148x_3_^2^ − 0.00096x_1_x_2_ − 0.00489x_1_x_3_	85.5
main oxidation product 1 of B3 (**3**) [%]	5.15	f(x) = 40.5 + 0.0190x_1_ − 0.0781x_2_ + 1.05x_3_ + 0.00609x_1_^2^ + 0.00011x_2_^2^ + 0.00809x_3_^2^ − 0.0383x_1_x_3_	78.1
main oxidation product 2 of B3 (**4**) [%]	3.75	f(x) = −66.5 + 1.90x_1_ + 0.146x_2_ + 1.86x_3_ − 0.00949x_1_^2^ − 0.00015x_2_^2^ − 0.0167x_3_^2^ − 0.00043x_1_x_2_ − 0.0178x_1_x_3_ − 0.00169x_2_x_3_	93.5
main oxidation product 1 of B4 (**5**) [%]	7.19	f(x) = 112.7 − 2.60x_1_ − 0.131x_2_ − 0.425x_3_ + 0.0210x_1_^2^ + 0.000154x_2_^2^ +0.0101x_3_^2^ − 0.00912x_1_x_3_ + 0.00102x_2_x_3_	92.8
main oxidation product 2 of B4 (**6**) [%]	4.57	f(x) = 0.600 + 1.31x_1_ + 0.140x_2_ + 0.684x_3_ − 0.00686x_1_^2^ − 0.000068x_2_^2^ − 0.00583x_3_^2^ − 0.00087x_1_x_2_ − 0.0210x_1_x_3_ − 0.00192x_2_x_3_	93.0

**Table 3:** Overview of the optimal reaction conditions x_1_, x_2_ and x_3_ as well as the optimisation of the formation rate of the oxidation products **1**–**6** determined theoretically and experimentally (n = 3).

Optimised Products	Retention Time (R_t_) [min]	x_1_ [°C]	x_2_ [min]	x_3_ [*n*/*n*]	95% Prediction Interval of Formation Rate [%]	Experimental Formation Rate [%]
procyanidin A1 (**1**) [%]	15.2	61.4	77.2	9:28.8	38.2–52.6	47.4 ± 1.14
procyanidin A2 (**2**) [%]	16.1	66.9	10.0	9:26.5	17.9–28.0	27.5 ± 0.76
main oxidation product 1 of B3 (**3**) [%]	5.15	25.0	10.0	9:50.0	48.0–89.3	48.6 ± 4.01
main oxidation product 2 of B3 (**4**) [%]	3.75	75.0	314.0	9:12.5	33.8–48.3	32.0 ± 1.14
main oxidation product 1 of B4 (**5**) [%]	7.19	25.0	10.0	9:12.5	40.7–66.9	45.0 ± 5.14
main oxidation product 2 of B4 (**6**) [%]	4.57	53.7	360.0	9:12.5	50.6–75.0	60.2 ± 3.68

## Data Availability

Data will be made available on request.

## Supplementary Materials

The following supporting information can be downloaded at: https://www.mdpi.com/article/10.3390/molecules30010111/s1, Figure S1: Typical MS^2^ fragmentation in negative ionisation mode of (**A**) procyanidin A1 and A2 according to Poupard et al. [30] and (**B**) the spiro-linked-compound of B3 (C-(4 → 8 → 6′)-C) based on Hibi and Yanase [20]; Figure S2: MS fragmentation of compound **1** and **3** with the different quinone methide cleavage fragmentations of the A-type PCs and the assumed spiro-linked-compounds of **3–6**; Figure S3: Pareto plots of the standardised effects with *α* = 0.05 (x_1_ = temperature, x_2_ = time, x_3_ = ratio B-type to DPPH radical) for the formation rate of the main oxidation products **1–6**: (**A**) procyanidin A1 (**1**), (**B**) procyanidin A2 (**2**), (**C**) oxidation product 1 of B3 (**3**), (**D**) oxidation product 2 of B3 (**4**), (**E**) oxidation product 1 of B4 (**5**) and (**F**) oxidation product 2 of B4 (**6**); Table S1: Mass spectra MS^n^ of the main products of the DPPH-induced oxidation of B1; Table S2: Mass spectra MS^n^ of the main products of the DPPH-induced oxidation of B2; Table S3: Mass spectra MS^n^ of the main products of the DPPH-induced oxidation of B3; Table S4: Mass spectra MS^n^ of the main products of the DPPH-induced oxidation of B4.

## References

[1] Gu L., Kelm M.A., Hammerstone J.F., Beecher G., Holden J., Haytowitz D., Gebhardt S., Prior R.L. (2004). Concentrations of proanthocyanidins in common foods and estimations of normal consumption. J. Nutr..

[2] Haslam E., Harborne J.B., Mabry T.J. (1982). Proanthocyanidins. The Flavonoids: Advances in Research.

[3] Oliveira J., Mateus N., de Freitas V., Ramawat K.G., Mérillon J.-M. (2013). Flavanols: Catechins and Proanthocyanidins. Natural Products.

[4] Appeldoorn M.M., Sanders M., Vincken J.-P., Cheynier V., Le Guernevé C., Hollman P.C., Gruppen H. (2009). Efficient isolation of major procyanidin A-type dimers from peanut skins and B-type dimers from grape seeds. Food Chem..

[5] Esatbeyoglu T., Wray V., Winterhalter P. (2010). Dimeric procyanidins: Screening for B1 to B8 and semisynthetic preparation of B3, B4, B6, and B8 from a polymeric procyanidin fraction of white willow bark (*Salix alba*). J. Agric. Food Chem..

[6] Zhang H., Yerigui, Yang Y., Ma C. (2013). Structures and antioxidant and intestinal disaccharidase inhibitory activities of A-type proanthocyanidins from peanut skin. J. Agric. Food Chem..

[7] Liu L., Xie B., Cao S., Yang E., Xu X., Guo S. (2007). A-type procyanidins from Litchi chinensis pericarp with antioxidant activity. Food Chem..

[8] Alejo-Armijo A., Glibota N., Frías M.P., Altarejos J., Gálvez A., Salido S., Ortega-Morente E. (2018). Synthesis and Evaluation of Antimicrobial and Antibiofilm Properties of A-Type Procyanidin Analogues against Resistant Bacteria in Food. J. Agric. Food Chem..

[9] Lu Z., Jia Q., Wang R., Wu X., Wu Y., Huang C., Li Y. (2011). Hypoglycemic activities of A- and B-type procyanidin oligomer-rich extracts from different Cinnamon barks. Phytomedicine.

[10] Xie C., Wang K., Liu X., Liu G., Hu Z., Zhao L. (2023). Characterization and bioactivity of A-type procyanidins from litchi fruitlets at different degrees of development. Food Chem..

[11] Dong X.-Q., Zou B., Zhang Y., Ge Z., Du J., Li C. (2013). Preparation of A-type proanthocyanidin dimers from peanut skins and persimmon pulp and comparison of the antioxidant activity of A-type and B-type dimers. Fitoterapia.

[12] Kraus G.A., Yuan Y., Kempema A. (2009). A convenient synthesis of Type A procyanidins. Molecules.

[13] Burger J.F., Kolodziej H., Hemingway R.W., Steynberg J.P., Young D.A., Ferreira D. (1990). Oligomeric flavanoids. Part W. base-catalyzed pyran rearrangements of procyanidin B-2, and evidence for the oxidative transformation of B- to A-type procyanidins. Tetrahedron.

[14] Nonaka G., Morinoto S., Kinjo J., Nohara T., Nishioka I. (1987). Tannins and Related Compounds. L. Structures of Proanthocyanidin A-1 and Related Compounds. Chem. Pharm. Bull..

[15] Kondo K., Kurihara M., Fukuhara K., Tanaka T., Suzuki T., Miyata N., Toyoda M. (2000). Conversion of procyanidin B-type (catechin dimer) to A-type: Evidence for abstraction of C-2 hydrogen in catechin during radical oxidation. Tetrahedron Lett..

[16] Osman A.M., Wong K. (2007). Laccase (EC 1.10.3.2) catalyses the conversion of procyanidin B-2 (epicatechin dimer) to type A-2. Tetrahedron Lett..

[17] Chen L., Yuan P., Chen K., Jia Q., Li Y. (2014). Oxidative conversion of B- to A-type procyanidin trimer: Evidence for quinone methide mechanism. Food Chem..

[18] Jing S.-X., McDermott C.M., Flanders P.L., Reis-Havlat M., Chen S.-N., Bedran-Russo A.K., McAlpine J.B., Ambrose E.A., Pauli G.F. (2024). Chemical Transformation of B- to A-type Proanthocyanidins and 3D Structural Implications. J. Nat. Prod..

[19] Hayashi S., Nakano K., Yanase E. (2018). Investigation of color-deepening phenomenon in catechin-(4→8)-dimer as a proanthocyanidin model and structural determination of its derivatives by oxidation. Food Chem..

[20] Hibi Y., Yanase E. (2019). Oxidation of procyanidins with various degrees of condensation: Influence on the color-deepening phenomenon. J. Agric. Food Chem..

[21] Kataoka H., Kakumu Y., Agbo D.O., Taniguchi T., Yanase E. (2024). Computational study on the conformational flexibility-mediated intramolecular oxidative spirocyclization of procyanidin B4. J. Org. Chem..

[22] Suwa Y., Yanase E. (2022). Structure determination and formation mechanism of procyanidin B2 oxidation products. Tetrahedron.

[23] Soravia S., Orth A., Bohnet M. (2003). Design of Experiments. Ullmann’s Encyclopedia of Industrial Chemistry.

[24] Ferreira S.L.C., Bruns R.E., Ferreira H.S., Matos G.D., David J.M., Brandão G.C., Da Silva E.G.P., Portugal L.A., dos Reis P.S., Souza A.S. (2007). Box-Behnken design: An alternative for the optimization of analytical methods. Anal. Chim. Acta.

[25] Park H.-J., Kim S.-Y., Song N.-Y., Cho J.-G., Kang J.-H., Jeong T.-S., Lee D.-Y., Kim G.-S., Kim Y.-B., Kang H.-C. (2014). Procyanidins from the stem wood of Machilus japonica and their inhibitory effect on LDL oxidation. Arch. Pharm. Res..

[26] Killday K.B., Davey M.H., Glinski J.A., Duan P., Veluri R., Proni G., Daugherty F.J., Tempesta M.S. (2011). Bioactive A-type proanthocyanidins from Cinnamomum cassia. J. Nat. Prod..

[27] Alejo-Armijo A., Salido S., Altarejos J.N. (2020). Synthesis of A-Type Proanthocyanidins and Their Analogues: A Comprehensive Review. J. Agric. Food Chem..

[28] Yan F., Chen L., Chen W., Zhao L., Lu Q., Liu R. (2021). Protective effect of procyanidin A-type dimers against H2O2-induced oxidative stress in prostate DU145 cells through the MAPKs signaling pathway. Life Sci..

[29] Rue E.A., Rush M.D., van Breemen R.B. (2018). Procyanidins: A comprehensive review encompassing structure elucidation via mass spectrometry. Phytochem. Rev..

[30] Poupard P., Sanoner P., Baron A., Renard C.M.G.C., Guyot S. (2011). Characterization of procyanidin B2 oxidation products in an apple juice model solution and confirmation of their presence in apple juice by high-performance liquid chromatography coupled to electrospray ion trap mass spectrometry. J. Mass Spectrom..

[31] De Taeye C., Caullet G., Eyamo Evina V.J., Collin S. (2017). Procyanidin A2 and Its Degradation Products in Raw, Fermented, and Roasted Cocoa. J. Agric. Food Chem..

[32] Ito R., Yammamoto A., Kodama S., Kato K., Yoshimura Y., Matsunaga A., Nakazawa H. (2003). A study on the change of enantiomeric purity of catechins in green tea infusion. Food Chem..

[33] Esatbeyoglu T., Winterhalter P. (2010). Preparation of dimeric procyanidins B1, B2, B5, and B7 from a polymeric procyanidin fraction of black chokeberry (*Aronia melanocarpa*). J. Agric. Food Chem..

