# Supplementary material for: A Design of Experiments Approach to the Radical-Induced Oxidation of Dimeric C4-C8 Linked B-Type Procyanidins
Source: Molecules. 2024 Dec 30;30(1):111. doi: 10.3390/molecules30010111 (PMC11721415; doi:10.3390/molecules30010111)
Supplement: Supplementary file 1 [file molecules-30-00111-s001.zip › molecules-3390567-supplementary.pdf]

A Design of Experiment Approach to the Radical-Induced Oxidation of Dimeric C4-C8 Linked B-Type Procyanidins

Annik Fischer [1], Recep Gök [2] and Tuba Esatbeyoglu [1*]

[1] Department of Molecular Food Chemistry and Development, Institute of Food and One Health, Leibniz University Hannover, 30167 Hannover, Germany

[2] Institute of Food Chemistry, Technische Universität Braunschweig, 38106 Braunschweig, Germany

***Corresponding author:**

Prof. Dr. Tuba Esatbeyoglu

Department of Molecular Food Chemistry and Development
Institute of Food and One Health
Gottfried Wilhelm Leibniz University Hannover

Am kleinen Felde 30
30167 Hannover, Germany
Tel: +49-511-762-5589, Fax: +49-511-762-4927
Email: esatbeyoglu@foh.uni-hannover.de

e-mails:

Tuba Esatbeyoglu= esatbeyoglu@foh.uni-hannover.de

Annik Fischer= fischer@foh.uni-hannover.de

Recep Gök= r.goek@tu-braunschweig.de

ORCID:

T. Esatbeyoglu: 0000-0003-2413-6925

A. Fischer: 0000-0002-1977-3949

R. Gök: 0000-0002-1231-8679

Supplementary material:

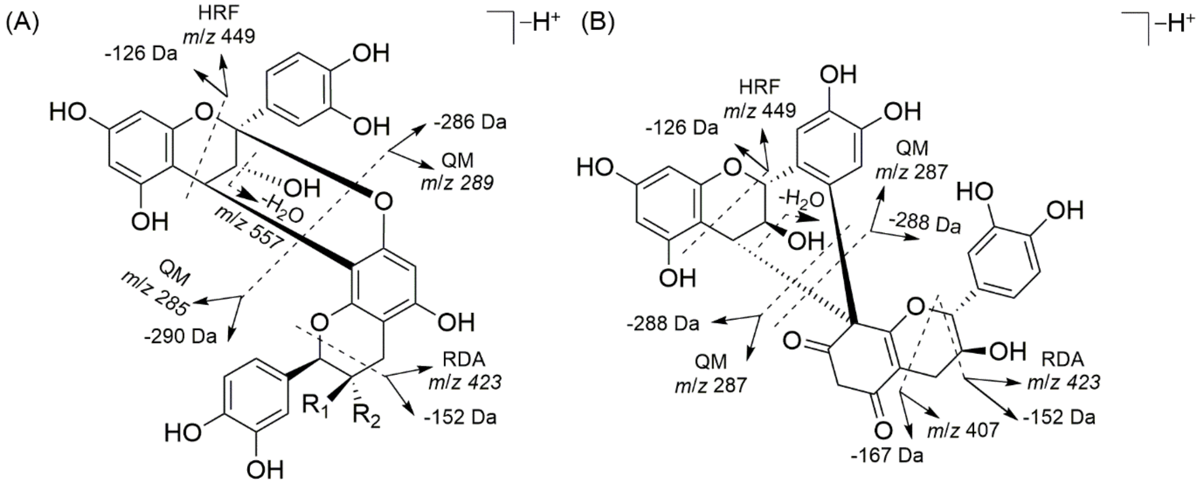

procyanidin A1: R_1= H, R_2= OH
procyanidin A2: R_1= OH, R_2= H

postulated spiro-linked-product of B3: C-(4→8→6')-C

Figure S1. Typical MS^2 fragmentation in negative isonisation mode of (**A**) procyanidin A1 and A2 according to Poupard et al. [1] and (**B**) the postulated spiro-linked-compound of B3 (C-(4→8→6')-C) based on Hibi and Yanase [2].

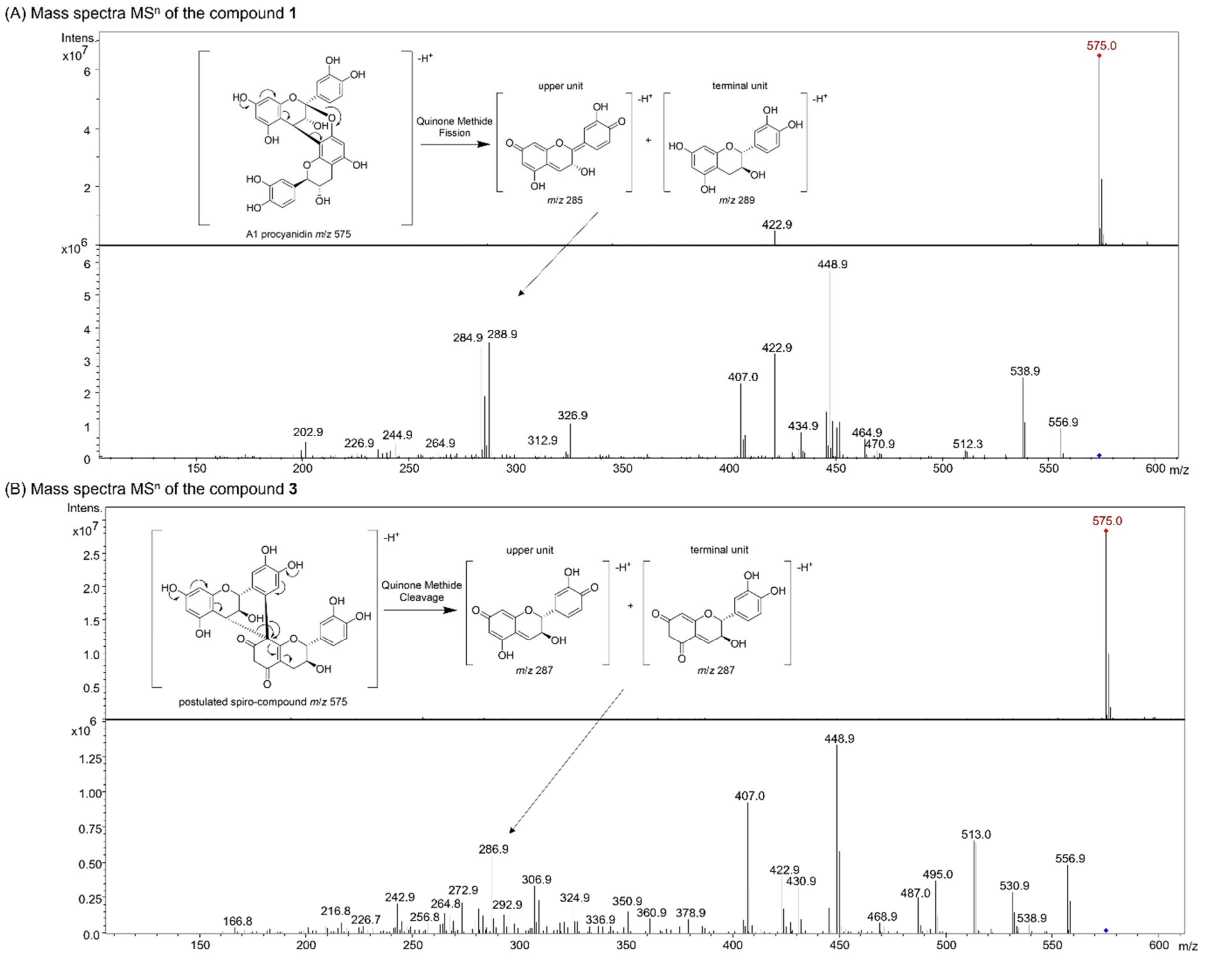

Figure S2. MS fragmentation of compound **1** (**A**) and **3** (**B**) with the different quinone methide cleavage fragmentations of the A-type PCs and the assumed spiro-linked-compounds of **3** – **6**.

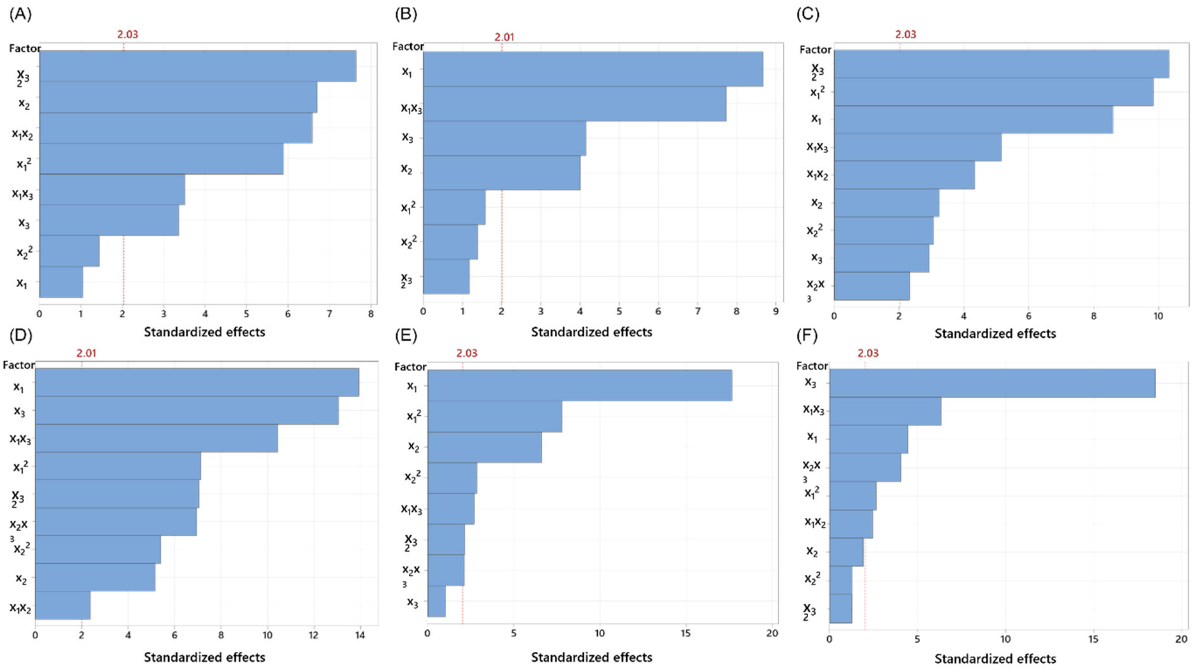

Figure S3. Pareto plots of the standardised effects with α= 0.05 (x_1= temperature, x_2= time, x_3= ratio B-type to DPPH radical) for the formation rate of the main oxidation products **1** – **6**: (**A**) procyanidin A1 (**1**), (**B**) procyanidin A2 (**2**), (**C**) oxidation product 1 of B3 (**3**), d) oxidation product 2 of B3 (**4**), (**E**) oxidation product 1 of B4 (**5**) and (**F**) oxidation product 2 of B4 (**6**).

Table S1. Mass spectra MS^n of the main products of the DPPH-induced oxidation of B1.

R_t [min]	$[M-H]^-$ [*m/z*]	MS^2 fragments [*m/z*]	Mass spectra MS^n
6.95	573	555, 511, 471, 392, 365, 347, 337	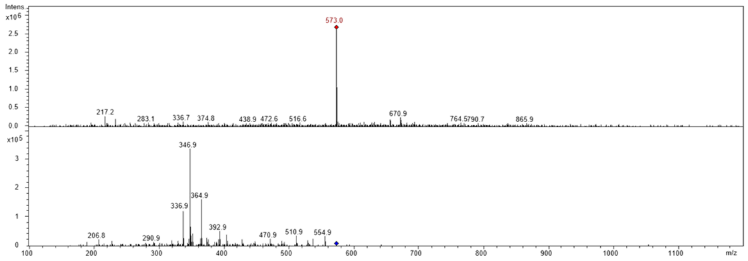
11.9	573	555, 447, 435, 389, 371, 285, 243, 201	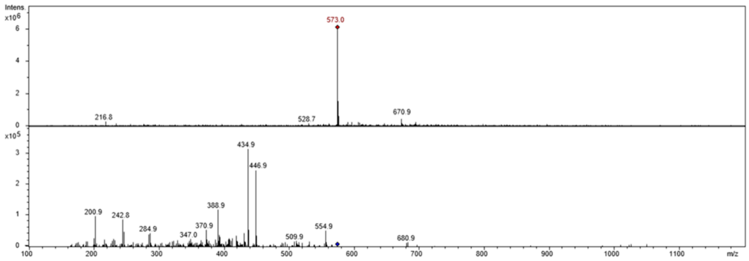
12.3	573	555,512, 447, 435, 391, 345, 285, 243, 201	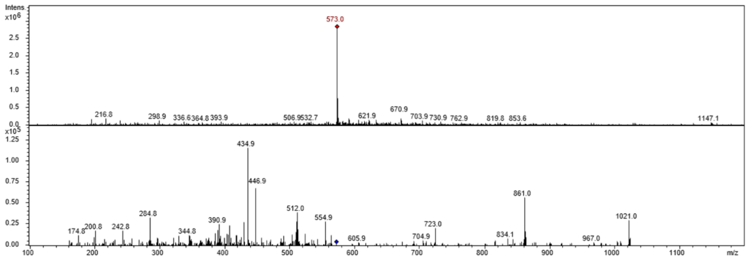
15.2 (**1**)	575	557, 539, 449, 423, 407, 327, 289, 285, 245	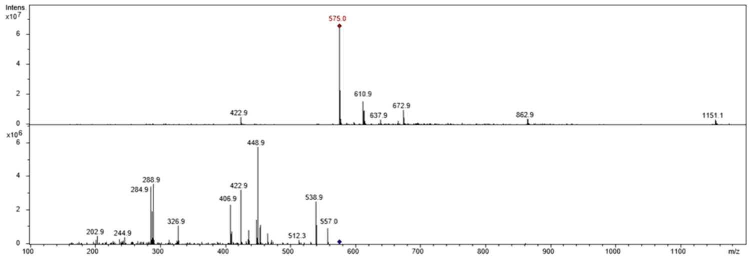
16.1	575	557, 539, 449, 423, 407, 289, 285, 245	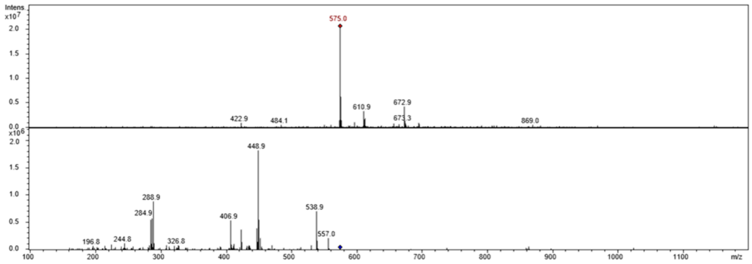

Table S2. Mass spectra MS^n of the main products of the DPPH-induced oxidation of B2.

R_t [min]	$[M-H]^-$ [*m*/*z*]	MS^2 fragments [*m*/*z*]	Mass spectra MS^n
4.55	573	555, 511, 471, 393, 365, 347, 337, 319	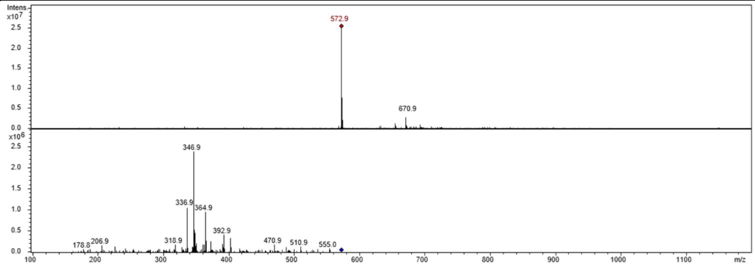
9.09	573	555, 449, 435, 417, 373, 317, 283, 229	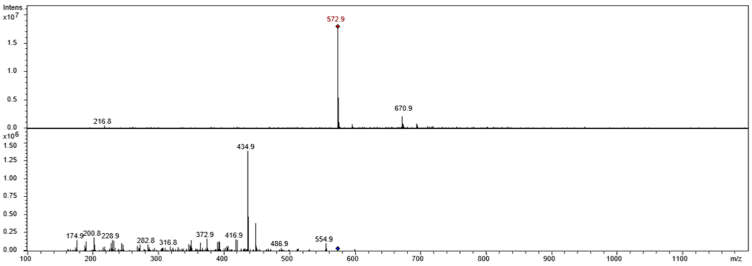
15.2	575	557, 539, 449, 423, 289, 285, 257	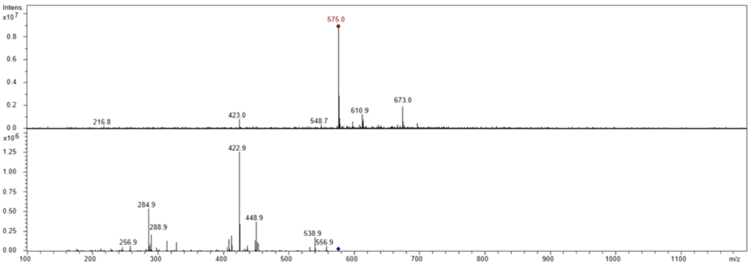
16.1 (**2**)	575	557, 539, 449,423, 327, 289, 285, 245	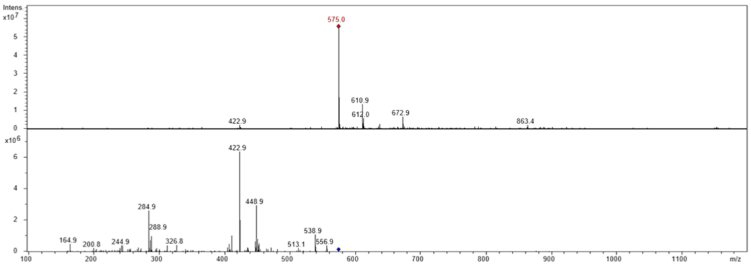

Table S3. Mass spectra MS^n of the main products of the DPPH-induced oxidation of B3.

R_t [min]	[M-H]$^-$ [*m*/*z*]	MS^2 fragments [*m*/*z*]	Mass spectra MS^n
3.75 (**4**)	575	557, 513, 495, 449,431, 423, 407, 379, 351, 287, 243	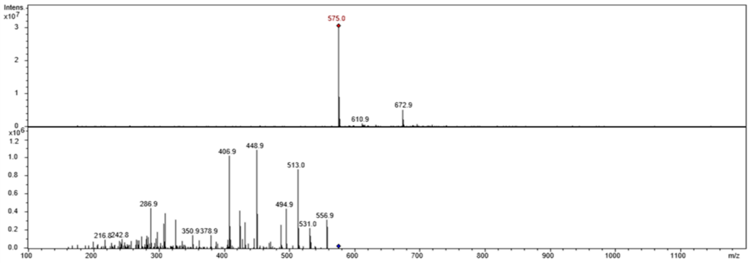
5.15 (**3**)	575	557, 513, 495, 449, 431, 423, 407, 351, 307, 287, 243	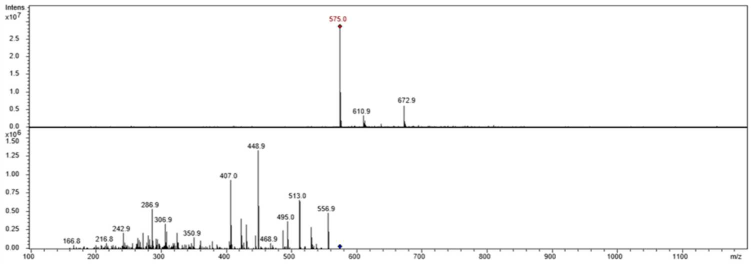
7.45	575	557, 513, 487, 423, 407, 379, 297	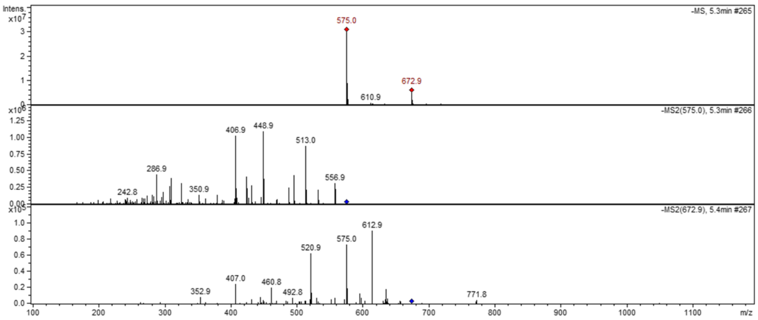
12.6	575	557, 531, 513, 487, 449, 431, 423, 407, 351, 325, 307, 287	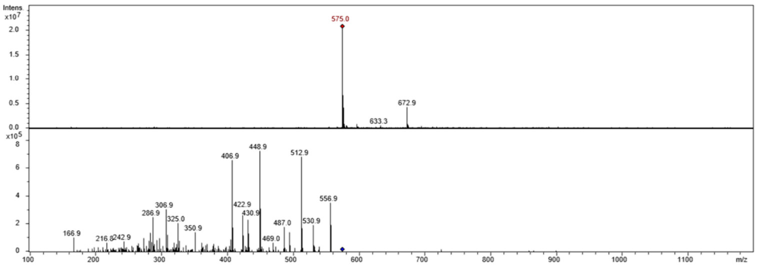
13.6	575	557, 531, 513, 487, 449, 407, 351, 325, 307, 287	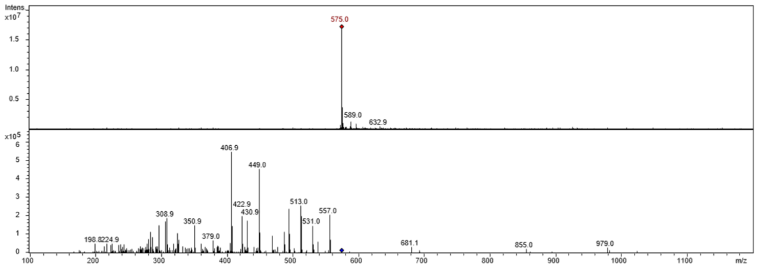

Table S4. Mass spectra MS^n of the main products of the DPPH-induced oxidation of B4.

R_t [min]	[M-H]$^-$ [*m*/*z*]	**MS^2 fragments [*m*/*z*]**	**Mass spectra MS^n**
4.57 (**6**)	575	557, 531, 513, 449, 423, 407, 379, 325, 287	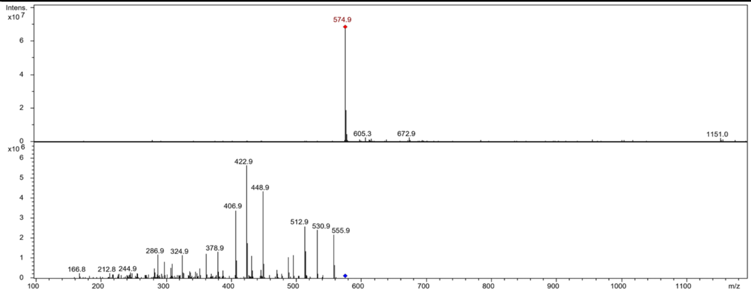
6.15	575	557, 531, 513, 487, 449, 423, 407, 361, 297	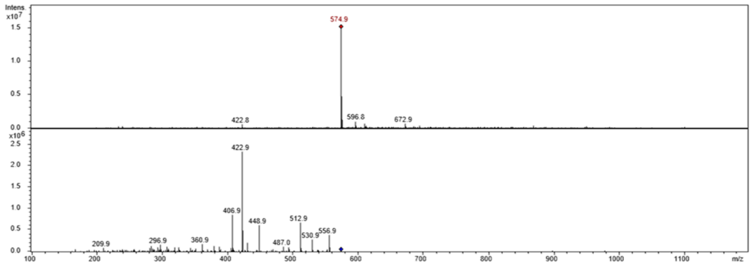
.19 (**5**)	575	557, 531, 513, 449, 423, 407, 379, 351, 287	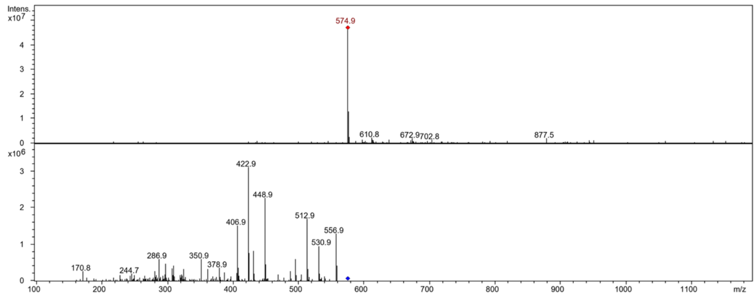
8.09	575	557, 531, 513, 449, 423, 407, 379, 325, 257	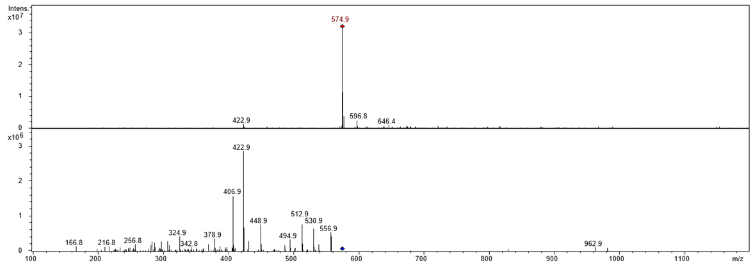
8.51	575	557, 531, 487, 449, 423, 407, 387, 361, 309	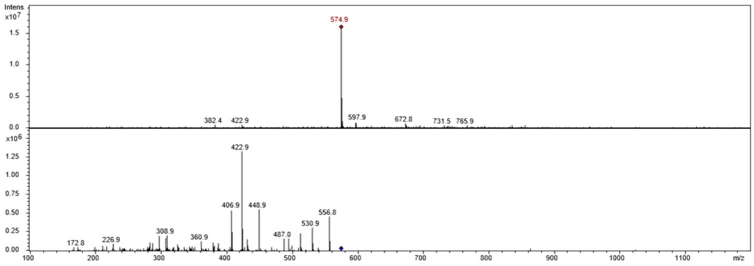

Rt [min]	[M-H]⁻ [*m*/*z*]	**MS² fragments [*m*/*z*]**	**Mass spectra MSⁿ**
10.2	575	557, 531, 513, 449, 423, 407, 387, 307, 248	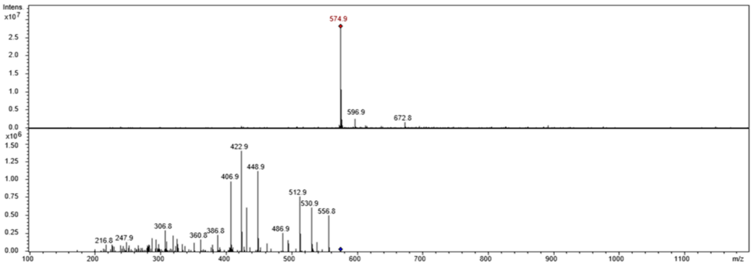
12.6	575	557, 531, 487, 449, 423, 407, 379, 351, 307, 245	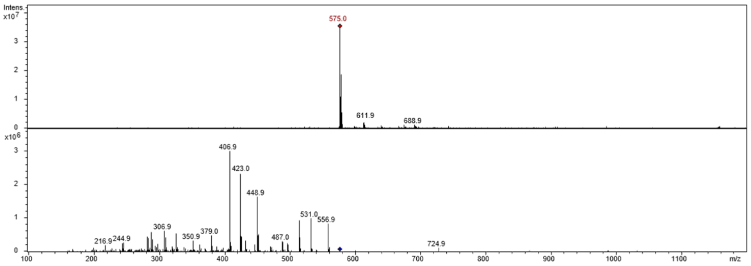

References

1. Poupard, P.; Sanoner, P.; Baron, A.; Renard, C.M.G.C.; Guyot, S. Characterization of procyanidin B2 oxidation products in an apple juice model solution and confirmation of their presence in apple juice by high-performance liquid chromatography coupled to electrospray ion trap mass spectrometry. *J. Mass Spectrom.* ***2011***, 46, 1186–1197, doi:10.1002/jms.2007.
2. Hibi, Y.; Yanase, E. Oxidation of Procyanidins with Various Degrees of Condensation: Influence on the Color-Deepening Phenomenon*. J. Agric. Food Chem.* **2019**, *67*, 4940–4946, doi:10.1021/acs.jafc.9b02085.
